# Educating to Tolerance: Effects of Communicating Social Psychology Research Findings

**DOI:** 10.5964/ejop.v11i3.888

**Published:** 2015-08-20

**Authors:** Francesco La Barbera

**Affiliations:** a Department of Political Sciences, University of Naples Federico II, Naples, Italy; Aalborg University, Aalborg, Denmark

**Keywords:** education to tolerance, ingroup bias, motivation, intergroup relations, communication

## Abstract

The effect of communicating social psychology research findings on ingroup bias in a classroom setting has been investigated. Two hundred and twenty one high school students either read or did not read a brief report about three classical social psychological studies, then completed evaluation scales for the ingroup and the outgroup. Participants’ motivation was manipulated, and the messages were different as regards the congruency between the content and participants’ actual intergroup experience. Results showed that communication exerted a significant effect in reducing ingroup bias for participants in the high motivation/high congruency condition, that is, the communication effect was moderated by the individual’s level of motivation and the content of the arguments proposed in the report. Practical implications of results for education work and stereotype change, limitations of the study, as well as possible directions for future research are discussed.

Ingroup bias is one of the most studied topics in social psychology: It is the individuals’ systematic tendency to identify with the group to which they belong and evaluate it (the ingroup) more positively than the group to which they do not feel they belong or identify with (the outgroup), specially on attributes which are relevant to the ingroup; this process represents the basis for stereotypes and prejudice (Brown, 1995; Tajfel & Turner, 1979). Several interpretations of the ingroup bias have been proposed: Tajfel (1981, 1982) highlighted that enhancing the ingroup compared to the outgroup protects the self-concept of the ingroup members, as well as their “subjective location in the social networks of which they are a part” (1982, p. 31), whereas Sherif (1967) underlined the importance of intergroup competition and lack of resources, which create a negative interdependence between groups (see also Brewer, 2000). Hamilton and Gifford (1976) focused upon cognitive processes which determine illusory correlation between undesirable behaviors and minorities.

A number of studies have explored different strategies for the reduction of ingroup bias, with different approaches and results (see Fiske, 2000, for a review). The aim of the current research is to investigate whether the communication of social psychology research findings about ingroup bias, intergroup conflict and stereotypes could be an effective educational strategy for reducing the ingroup bias: the basic idea is that a better understanding of the processes underpinning ingroup bias could promote its reduction. This simple concept seems to be implicit in teaching social psychology, yet it has not been sufficiently investigated by empirical research (for an exception, see Capozza, Volpato, & Falvo, 2003). Nonetheless, the hypothesis that educational processes based on social psychology research could improve understanding and tolerance, if supported by data, may have a huge practical impact, for example in relation to school multicultural programs and adults’ education programs against prejudice.

Several scholars have focused on the possibility of changing negative stereotypes of the outgroup through communication, thus reducing also ingroup/outgroup discrimination. This research line has a long tradition in social psychology, starting with Lewin (1951)’s seminal studies on changing attitudes, and has highlighted some basic points. According to Lewin, any stereotype change involves a dynamic process of de-learning and re-learning in a multidimensional restructuring process that may imply a significant level of difficulty for individuals. Therefore, people generally try to reject the counter-stereotypic information (Johnston, 1996), except for in some conditions like high motivation (Moreno & Bodenhausen, 1999). The crucial role of motivation has been also highlighted by the so-called two-path models of persuasion (Eagly & Chaiken, 1993; Petty & Cacioppo, 1986), whose validity has been confirmed also in the field of the stereotype change (Johnston & Coolen, 1995): when individuals are highly motivated, it is more likely that they carefully elaborate the message proposed, and the quality of arguments, which can be more or less convincing to them.

Therefore, current experiment investigated if high motivation in elaborating the message could increase the effect of communicating the findings of social psychological research on ingroup bias. In addition, we explored the effect of the quality of the arguments proposed by research findings; more specifically, we explored the effect of the *congruency* between the content of communication and the actual intergroup experience of participants.

What we should expect when the content of the research we present to participants seems to reflect quite directly their real intergroup experience? We suggest that the content of the message should be more convincing when it recalls the peculiarities of individuals’ actual intergroup relations and social context. On the contrary, if people feel the research rationale and findings as very far and different from the actual condition they live in, this could lead to their closure and refusal of the communication message.

This is in line with the traditional research on the self-reference effect (Kuiper & Rogers 1979; Markus 1977, 1980), which is the individuals’ association of self-relevant incoming information with information previously stored in memory (self-concept). Individuals who self-relate information are more likely to remember it, and to respond to it in a positive way. Consequently, self-reference can play an important role in a persuasion context (Cacioppo & Petty, 1979).

In our experiment, we investigated whether communication about a realistic intergroup conflict for incompatible goals would be more persuasive than other forms of communication, to an audience who had previous experience of such a conflict.

Finally, according to dual-path models of persuasion (Eagly & Chaiken, 1993; Petty & Cacioppo, 1986), only highly motivated individuals should focus their attention on the message; therefore, we also expected a significant interaction between participants’ motivation (which was manipulated in the experiment) and the content of arguments proposed.

In sum, the aim of our research was to investigate the effect that the communication of social psychology research findings had on ingroup bias, as a function of participants’ motivation, of the content of arguments, and of the interaction between the last two factors.

## Method

### Sample

We selected the intergroup relation between Italians and Chinese, which has been already studied by La Barbera and Cariota Ferrara (2010) in the same Italian district in which the present experiment has been carried out. In the last decades this area has been the target of a huge immigration which entailed deep social and economic changes. The Chinese immigrants, a very large group, have been in competition with locals in the textile area trade and for the acquisition of real properties. Chinese have been found, indeed, to be highly discriminated against and perceived as competitors by the locals (La Barbera & Cariota Ferrara, 2010).

Two hundreds and twenty-one high school students (151 females), took part to the experiment (age range: 15-19 years; *M* = 17.03; *SD* = 0.85). Their participation was voluntary and they completed the questionnaires (see below) in the classroom. The high schools were selected in the district just described.

### Materials

We selected descriptions of three well-known social psychological studies, which gave different explanations of the ingroup bias.

According to Tajfel, Billig, Bundy, and Flament (1971), the social categorization is the necessary and sufficient condition for the ingroup bias to emerge. Tajfel’s (1982) CIC approach (social Categorization, social Identity, social Comparison) states that individuals place themselves and others in social categories, which become part of their social identity; hence, they engage in a series of social comparisons from which they try to make sure that the ingroup proves better than the outgroup. In this way, they protect their own social identity and enhance their self-esteem, which depends on the groups they belong. Tajfel et al. (1971) randomly assigned their experimental subjects into two different groups, apparently on the basis of the result of a categorization task. After that, they asked participants to allocate resources to a member of the ingroup and a member of the outgroup. The main allocation strategy used by participants was the *maximum difference*, that is, trying to achieve the maximum difference between the two members (in favor of the ingroup one), irrespective of the total amount given to the ingroup and outgroup members.

The second description was based on the work of Sherif (1967), who claimed that individuals’ favouritism for their ingroup should be determined by the presence of incompatible goals, which create competition between groups for gaining resources. In the Robbers Cave Experiment, Sherif and colleagues, in a summer camp setting, created two groups of boys from middle-class families, then made them compete in games and challenges. Participants were told that, at the end of the camp, each member of the winning group was about to win a pocket knife. This competition had a negative effect on intergroup relations. Then Sherif and colleagues tried to reduce the animosity between groups, showing that superordinate goals (which require the cooperation of more than one group to be achieved) reduced conflict significantly. Sherif’s conclusion was that intergroup conflict is based upon real competition for incompatible goals, which create a negative interdependence between groups, whereas superordinate goals, promoting a positive interdependence, foster positive relations.

The final description was of the work of Hamilton and Gifford (1976), who proposed that biased evaluations towards the ingroup vis-a-vis an outgroup could be determined by cognitive errors in processing information. Indeed, in an experiment on illusory correlation, participants read a series of sentences describing either desirable or undesirable behaviors, which were attributed to the members of two different groups. Most of the sentences were associated with Group A, and the remaining few were associated with Group B. Each group had the same proportions of positive and negative behaviors, so there was no real association between behaviors and group membership. Nonetheless, participants significantly overestimated the association between the less frequent and undesirable behaviors and the minority group members. Therefore, in a context in which there is a majority group (e.g. the locals) and a minority group (e.g. the immigrants), one could expect the majority group members to make an illusory correlation between undesirable behaviors and minority groups, thus supporting negative stereotype of the outgroup.

### Procedure

1 – In a questionnaire, participants were told that they would be asked to read a well-known scientific study about intergroup relations. In the “high motivation” condition, participants were told that subsequently they would have to give their evaluation about “two groups that live in Italy”, with the indication of the groups they would have to assess (Italians and Chinese). In the “low motivation” condition we gave no anticipation of this.

2 – Each participant either read one of the three research descriptions, or read nothing; therefore, there were three experimental conditions in the study (Hamilton vs. Tajfel vs. Sherif), and a control condition in which participants read no research description. Then, an item asked participants if they already had knowledge of the research; the answers always were negative.

3 – Participants’ ingroup bias was measured by an instrument we built through a pilot-study: 43 students of another high school of the same district completed a free association task; with the six adjectives that most frequently have been associated to both “Italians” and “Chinese” stimuli, we built 7-point semantic differential scales (Osgood, Suci, & Tannenbaum, 1957): “desirable/undesirable”, “unpleasant/pleasant”, “beautiful/ugly”, “sunny/dark”, “clean/dirty”, “kind/unkind”. The reliability of the scale was satisfactory for both Italians, α = .70, and Chinese, α = .83. An average score of the six items was created, with higher values corresponding to more negative evaluation of the target group.

Each participant evaluated the two target groups (Italians and Chinese) on these six dimensions. The order of presentation of the target group was counterbalanced. Finally, participants were fully debriefed and thanked for participation in the study.

Hence, the experimental design is defined by two between participants factors (Message: Hamilton vs. Tajfel vs. Sherif vs. No message; Motivation: High vs. Low) and one within participants factor (Target-group: Italians vs. Chinese). Participants were randomly attributed to one of the the eight experimental conditions.

## Results

A new variable was created for measuring ingroup bias^i^ by subtracting participants’ evaluation of Italians from their evaluations of Chinese (higher values correspond to higher ingroup bias).

To examine the effects of message, motivation, and their interaction on ingroup bias, we carried out an ANOVA with two between participants factors, motivation (High vs. Low) and message (Sherif vs. Tajfel vs. Hamilton vs. Control), with ingroup bias as the dependent variable. We found a marginal effect of motivation, *F*(7, 213) = 3.23, *p* = 0.07, η^2^ = 0.015): ingroup bias scores were slightly higher in the high motivation condition (*M* = 2.37, *SD* = 1.36) than in the low motivation condition (*M* = 1.79, *SD* = 1.33). On the other hand, there was no significant main effect of the message, *F*(7, 213) = 1.73, *p* = 0.16. The effect of the crucial interaction of motivation by message was statistically significant, *F*(7, 213) = 3.85, *p* = 0.01, η^2^ = 0.051. Means and standard deviations of participants’ ingroup bias over conditions are provided in Table 1.

**Table 1:** Ingroup Bias Against Chinese

Message	High motivation			Low motivation		
	*M*	*SD*	*N*	*M*	*SD*	*N*
Sherif	2.05^a^	1.22	28	2.19^c^	1.24	30
Tajfel	1.96^a^	1.40	26	1.81^cd^	1.28	28
Hamilton	2.48^ab^	1.47	29	1.27^d^	1.23	26
No message	2.94^b^	1.20	28	1.82^cd^	1.49	26
Total	2.37	1.36	111	1.79	1.33	110

*Note*. Table shows mean scores and standard deviations of participants’ ingroup bias against Chinese outgroup over conditions. The means marked with different letters are different at *p* < 0.05 level.

Simple effects analysis showed that, in the high motivation condition, the effect of message type was significant, *F*(3, 107) = 2.68, *p* = 0.05, η^2^ = 0.070: participants’ ingroup bias scores in the Tajfel and Sherif conditions are lower than those of participants in the control condition, *t*(52) = 2.40, *p* = 0.02, and *t*(54) = 2.62, *p* = 0.01, respectively, whereas there is no significant difference between participants’ scores in the Hamilton and control conditions, *t*(55) = 1.25, *p* = 0.22.

At low motivation level, the effect of message type was also significant, *F*(3, 106) = 2.88, *p* = 0.040, η^2^ = 0.075, but pairwise comparisons showed no significant difference between the three experimental conditions and the control condition (all *p*s > .10), that is, the ingroup bias of participants who read the research report was not significantly different from the ingroup bias of participants who read nothing. The only significant difference was found between Sherif and Hamilton conditions, *t*(54) = 3.02, *p* = 0.004, with those in the Sherif condition reporting high ingroup bias than those in the Hamilton condition.

## Discussion

The aim of the present research was to investigate the effect of using social psychology research findings for reducing ingroup bias in a classroom setting. Our hypothesis of a significant effect of the scientific communication moderated by participants’ motivation and quality of message was supported by the significant two-way interaction: the two between-participants factors interactively affected participants’ ingroup bias.

When participants read about social psychology research while knowing that they will be asked about group evaluations, it seems that the evocation of the target group increases the saliency of the intergroup comparison (Tajfel & Turner, 1979), which makes participants in the Sherif condition more motivated to elaborate the message; we argue that they then detected a high congruency between the real competition they live with Chinese and Sherif’s findings. Hence, motivation and congruency moderate the effect of the scientific communication in reducing the ingroup bias. In the low motivation condition, instead, we found no significant effect of scientific communication compared to the control condition^ii^: therefore, a high level of motivation seems to be a necessary (but not satisfactory) condition to the ingroup bias reduction through communicating social psychological research.

We found the same pattern of results in the case of participants who read Tajfel’s research, but not in the case of Hamilton and Gifford’s study. The positive effect of the message based on Tajfel’s research was unexpected. Nonetheless, both Sherif’s and Tajfel’s studies regard ingroup-outgroup confrontation for gaining resources. Although there are marked differences between the two studies as regards the theoretical background, results and general interpretation of the intergroup conflicts (see Tajfel & Turner, 1979), in both cases participants maybe have focused their attention on the aspect of intergroup competition for something real (a pocketknife in the case of Sherif’s experiment, a certain amount of money-points for Tajfel’s), in particular because they were biased in that way by the previous evocation of Italian vs. Chinese intergroup comparison. In other words, Tajfel’s experiment, showing people’s propensity to promote the ingroup in gaining resources (though in a minimal intergroup situation), may have been felt by participants as very close to the real situation in which they live with Chinese members of their town, differently from Hamilton and Gifford’s study, in which the issue of gaining resources is not present at all. This interpretation of results further underlines the importance of the content of scientific communication. Nonetheless, this is an *ex post* speculation which should be deepened in future studies, in particular because the current study does not provide any measure of the underlying mechanisms proposed.

In addition, a possible problem in the manipulation of motivation could be that participants who have been told they had to evaluate two groups after their reading of a study about intergroup bias (high motivation condition), thought they were expected to be more positive towards the outgroup. However, we only found a marginal and small-sized main effect of motivation on the ingroup bias scores, which were slightly higher in the high motivation condition than in the low motivation condition. Therefore, this concern seems to be somewhat contradicted by the empirical evidence.

In sum, social psychology-based scientific communication seems to have some potential in reducing ingroup bias, and its positive effects seem to be moderated by the individuals’ motivation and the content (congruency) of message, specifically, its congruency with life experiences. Hence, our results support the idea that educational paths based on social psychology knowledge might promote better intergroup relations; nonetheless, they also suggest that not all messages could be effective for all intergroup relations, and the attention paid to the specific situation and to the real context in which intergroup conflicts take place could be relevant (see Lewin, 1946). Therefore, it would be important to study in depth the relations between the content of communication and the peculiarities of the intergroup comparison which is the target of the educational work.

One limitation of the current study is that it considered a very specific social context. The positive effects of communicating social psychological research results should be replicated in studies with larger and more heterogeneous samples, also taking into account the multilevel nature of the research population (individuals, classrooms, schools, cities). Moreover, the current study gave no information on the stability of the ingroup bias reduction over time. This is a very important question, which could be deepened by longitudinal research. Future research could also fruitfully explore the effects of different educational strategies based on social psychology’s findings (e.g. written communications, lectures, discussions), also in action-research programs.

If confirmed, our results could open new promising ways for education towards tolerance. Lessons from social psychology research could be adequately introduced, for example, in the elementary, middle, and high school education programs, as a straightforward way to reduce and/or prevent prejudice among wide populations. In addition, social psychology-based strategies, such as the discussion of research findings related to ingroup bias and prejudice, could be used for specific adults’ education programs in the case of social context with actual problems of intergroup conflicts.
